# Supplementary figures and images for: Vegetative compatibility groups partition variation in the virulence of Verticillium dahliae on strawberry
Source: PLoS One. 2018 Feb 16;13(2):e0191824. doi: 10.1371/journal.pone.0191824 (PMC5815587; doi:10.1371/journal.pone.0191824)

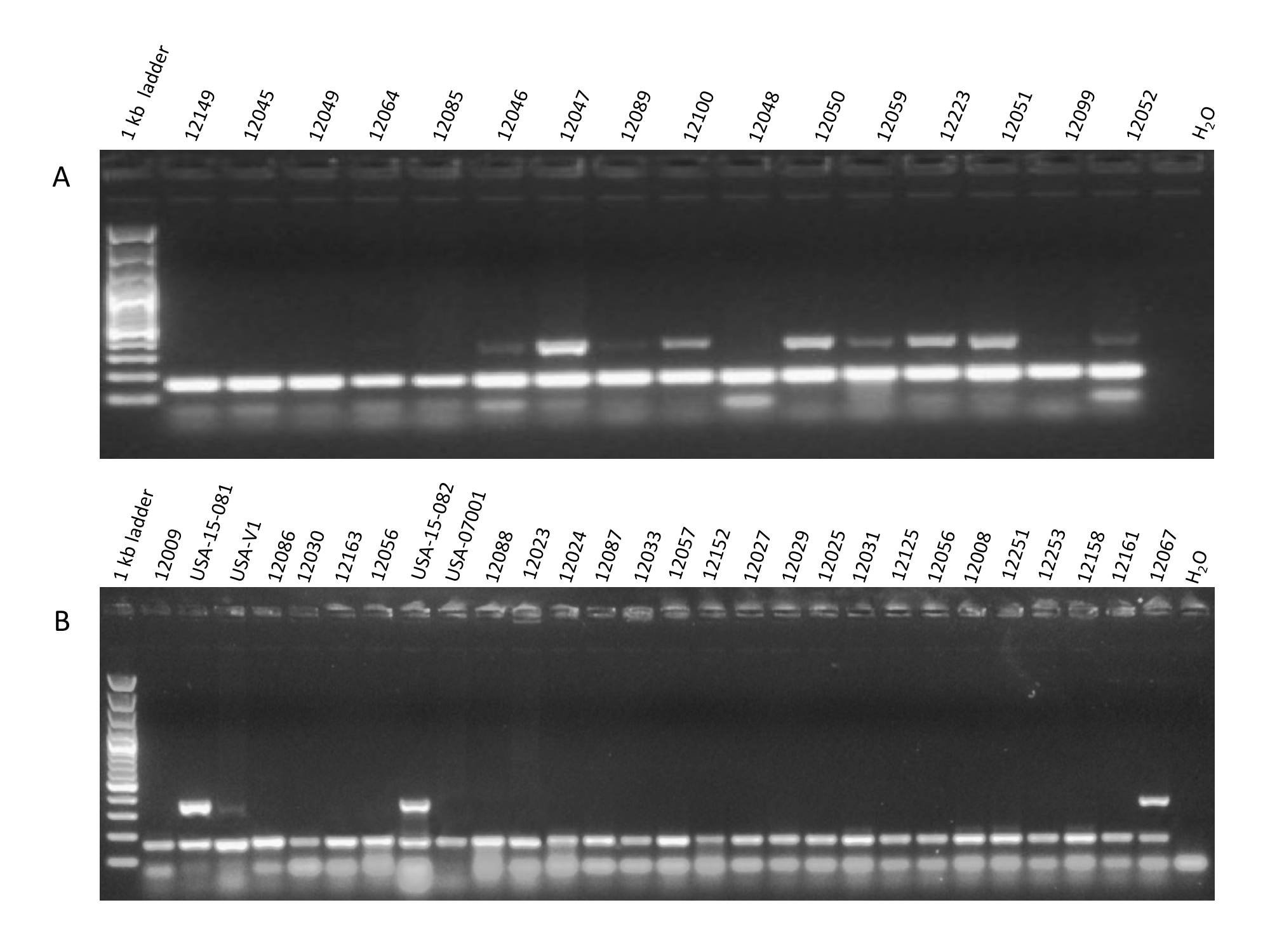

Supplement: S1 Fig — Isolates from UK hop, acer, raspberry, potato, phlox, cotinus, and chrysanthemum (A) and strawberries (B) from UK and California. Primers target for V. dahliae intergenic spacer regions were used as control for DNA quality. The isolate of 12067 is a ‘race 1’ isolate as positive control and NTC was negative control of sterilized water. (TIFF) [file pone.0191824.s001.tiff]
