# Supplementary material for: Vegetative compatibility groups partition variation in the virulence of Verticillium dahliae on strawberry
Source: PLoS One. 2018 Feb 16;13(2):e0191824. doi: 10.1371/journal.pone.0191824 (PMC5815587; doi:10.1371/journal.pone.0191824)
Supplement: S1 Table — (DOCX) [file pone.0191824.s002.docx]

**^S1^** **^Table. Primers used in this study.^**

| ^Primer^ | ^Sequence (5'–3')^ | ^Purpose^ |
| --- | --- | --- |
| ^VdAve1-F^ | ^CTTCACTGGTCACTGCCGAT^ | ^Determination of 1 strains in the UK.^ |
| ^VdAve1-R^ | ^CGATGTTGACCGCCTTATGC^ |  |
| ^NPAve1-F^ | ^ATGACCTAGUTATGCGAGCAGTTGTGAAG^ | ^Native promoter of Ave1 gene to construct Ave1 expression vector^ |
| ^NPAve1-R^ | ^ATACAGCCUTTCCGGGTAACTTGTAATT^ |  |
| ^Vd-F929-947^ | ^CGTTTCCCGTTACTCTTCT^ | ^Control of DNA quality in PCR targeting^ *^V. dahliae^* ^IGS (intergenic spacer ) region. Produce size = 160 bp^ |
| ^Vd-R1076-1094^ | ^GGATTTCGGCCCAGAAACT^ |  |
| ^VdAve1C-Fa^ | ^AGGCTGTAUGCCAATTACAAGTTACCCG^ | ^Amplification of the ORF (open reading frame) and terminator for construction of Ave1 expression vector^ |
| ^VdAve1C-Ra^ | ^ATTAAACCTUCCCCTAAAGCAATTTTGAT^ |  |
| ^Hyg-F^ | ^AGTCGGGGGATCCTCTAG^ | ^Validation of Ave1 knock-in mutants^ |
| ^Hyg-R^ | ^GGGCCCATCGATGATCAG^ |  |
| ^qAve1-F^ | ^ACCCACCCTACCTTCCCACT^ | ^Detection of Ave1 expression by qPCR. Efficiency = 0.962^ |
| ^qAve1-R^ | ^TCAACCACCCGCACATCA^ |  |
| ^btubulin-F^ | ^TGGCTACCTTCTCGGTCGTT^ | ^Housekeeping gene in qPCR.^  ^Efficiency = 0.998^ |
| ^btubulin-R^ | ^AGCTTGAGGGTGCGGATG^ |  |
| ^EF-F^ | ^TACAACCCCAAGACTGTCGC^ | ^Housekeeping gene in qPCR.^  ^Efficiency = 1.013^ |
| ^EF-R^ | ^CTTCCAGCCCTTGTACCAGG^ |  |
| ^DB19^ | ^CGGTGACATAATACTGAGAG^ | ^Vegetative compatibility group specific primers^ |
| ^DB22^ | ^GACGATGCGGATTGAACGAA^ |  |

^a^ Underlined text in primer sequences are junction primers following Sørensen et al (2014).
