# Supplementary material for: Vegetative compatibility groups partition variation in the virulence of Verticillium dahliae on strawberry
Source: PLoS One. 2018 Feb 16;13(2):e0191824. doi: 10.1371/journal.pone.0191824 (PMC5815587; doi:10.1371/journal.pone.0191824)
Supplement: S3 Table — SE is standard error. (DOCX) [file pone.0191824.s004.docx]

**S3 Table. Dunnett post hoc test on ANOVA to determine differences between disease scores of Hapil plants 6 weeks post inoculation with isolate 12008 and each transformant or ‘race 1’ isolate 12067.** SE is standard error.

| Transformant | Estimate | SE | t | *p* |
| --- | --- | --- | --- | --- |
| PA2 | 0.3333 | 0.762 | -0.437 | 0.973 |
| PA3 | 0.500 | 0.762 | 0.656 | 0.715 |
| PA4 | 0.667 | 0.762 | 0.875 | 0.617 |
| PA5 | 1.00 | 0.762 | 1.312 | 0.408 |
| PA6 | -1.67 | 0.762 | -2.187 | 1 |
| PG1 | 4.74 x 10^-15^ | 0.762 | 0 | 0.917 |
| PG2 | 5.00 | 0.762 | 0.656 | 0.715 |
| PG3 | 0.67 | 0.762 | 0.875 | 0.617 |
| PG4 | -0.33 | 0.762 | -0.437 | 0.973 |
| PG5 | -1.00 | 0.762 | -1.312 | 0.999 |
| 12067 | -2.17 | 0.762 | -2.844 | 1 |
