# Supplementary material for: Vegetative compatibility groups partition variation in the virulence of Verticillium dahliae on strawberry
Source: PLoS One. 2018 Feb 16;13(2):e0191824. doi: 10.1371/journal.pone.0191824 (PMC5815587; doi:10.1371/journal.pone.0191824)
Supplement: S4 Table — SE is standard error. (DOCX) [file pone.0191824.s005.docx]

**S4 Table. Dunnett post hoc test on ANOVA to determine differences between relative Ave1 expression of the ‘race 1’ isolate 12067 and each transformant.** SE is standard error.

| Transformant | Estimate | SE | t | *p* |
| --- | --- | --- | --- | --- |
| PA2 | -23.8 | 2.55 | -9.3 | < 1x10^-04^ |
| PA3 | -23.8 | 2.55 | -9.3 | < 1x10^-04^ |
| PA4 | 2.3 | 2.55 | 0.9 | 0.99 |
| PA5 | -19.8 | 2.55 | -7.8 | < 1x10^-04^ |
| PA6 | -24.6 | 2.55 | -9.6 | < 1x10^-04^ |
| PG1 | -7.6 | 2.55 | -3.0 | 0.01 |
| PG2 | -16.9 | 2.55 | -6.6 | < 1x10^-04^ |
| PG3 | -20.5 | 2.55 | -8.0 | < 1x10^-04^ |
| PG4 | -11.3 | 2.55 | -4.4 | 0.00 |
| PG5 | -17.5 | 2.55 | -6.9 | < 1x10^-04^ |
